# Susceptibility to Hyperglycemia in Rats With Stress-Induced Depressive-Like Behavior: Involvement of IL-6 Mediated Glucose Homeostasis Signaling

**DOI:** 10.3389/fpsyt.2020.00557

**Published:** 2020-06-23

**Authors:** Xiaojuan Li, Wenqi Qiu, Nan Li, Xiaoli Da, Qingyu Ma, Yajing Hou, Tingye Wang, Ming Song, Jiaxu Chen

**Affiliations:** ^1^Formula-pattern Research Center, School of Traditional Chinese Medicine, Jinan University, Guangzhou, China; ^2^School of Traditional Chinese Medicine, Beijing University of Chinese Medicine, Beijing, China

**Keywords:** depression, hyperglycemia, interleukin 6, glucose transporter 4, hypothalamus

## Abstract

Depression is a common psychiatric disorder comorbid with diabetes and may lead to high morbidity, disability, and mortality. However, the underlying mechanism behind their association remains unknown. Cytokine-mediated inflammation in brain may play important roles in the pathogenesis of depression and insulin resistance. In the present study, we subjected the rats to chronic unpredictable mild stress (CUMS) for 3 to 8 weeks. The tests to ascertain depression-like behaviors including open field test (OFT) and forced swimming test (FST) were performed, and levels of morning fasting blood glucose, triglyceride (TG), total cholesterol (CHOL), high density lipoprotein cholesterol (HDL-C), and low density lipoprotein cholesterol (LDL-C), body weight, food intake, histopathological examinations of liver, adipose tissues and hypothalamus, hypothalamic GLUT4 as well as the IL-6-mediated glucose homeostasis signaling pathway were measured. The results showed that CUMS exposure resulted in the depression-like behavior at various time points in rats. Moreover, the rats exhibited increased peripheral glucose levels, impaired hepatocytes and hippocampal neurons, and decreased hypothalamic GLUT4 levels after 6 weeks of CUMS exposure. Meanwhile, activated IL-6 but suppressed IL-6-mediated glucose homeostasis signaling was observed in the hypothalamus. Markers of lipid metabolism including TG, CHOL, HDL-C and LDL-C were dysregulated, and body weight and food intake were decreased in the CUMS-exposed rats. Our results show that depressed rats induced by 6-week CUMS stimulation display susceptibility to hyperglycemia, which is associated with IL-6-mediated inhibition of glucose homeostasis signaling in the hypothalamus.

## Introduction

Stress is commonly defined as a real or perceived threat to one’s safety (1). It can be categorized as “good stress”, “tolerable stress”, and “toxic stress”. Toxic stress refers to a situation in which a person chronically confronts adverse events that exceed his or her ability to cope with them effectively, resulting in adverse effects on the behavior and physiology of the person (2). Chronic stress generally evokes certain emotional and physiological reactions, and is one of the most important factors responsible for mental disorders in human beings (3). Unfortunately, depression is a highly prevalent chronic stress-induced psychiatric disorder but with limited treatment options and poorly understood pathophysiology. Behavioral impairment due to chronic stress also affects the systemic physiology and has been linked to the metabolic disorders such as diabetes and cardiovascular diseases (4). Emerging evidence demonstrated that depression could be an independent risk factor for the development of diabetes (5). Clinical data have also reported that one out of every four people suffering from type 2 diabetes mellitus (T2DM) also suffers from some extent of depression. In addition, depression also increases the risks of hyperglycemia, insulin resistance, and micro- and macrovascular complications (6). Although the impact of psycho-social stress on energy metabolism is increasingly being recognized, whether the depression-like behaviors induced by long-term chronic stress mediate an individual’s susceptibility or resilience to glucose homeostasis remains unknown and the molecular mechanisms underlying the relationship between chronic stress, depression, and glucose homeostasis are yet to be elucidated.

Chronic unpredictable mild stress (CUMS) is highly prevalent in several neuropsychiatric disorders such as depressive disorder in rodent models. CUMS-induced behavioral changes are intended to be homologous to depression, and thus can be used as an experimental tool in understanding the pathology of depression (7). When rats or mice are exposed to chronically mild but unpredictable stressors, several obvious behavioral changes such as decreased response to rewards, increased response to hopelessness, and decreased response to a novelty environment are observed. These behavioral changes correlate with the core symptoms of depression such as anhedonia, despair, and loss of interest, respectively. The CUMS model is commonly used for assessment of antidepressant effects of various therapeutic interventions (8–10). Notably, emerging evidence indicates that animals exposed to chronic stress exhibit metabolic abnormalities including insulin resistance, glucose intolerance, and hyperlipidemia (11), which is in agreement with the clinical reports that stress related depression is highly comorbid with diabetes. However, the effect of CUMS on peripheral glucose levels and its potential mechanism have largely been unexplored.

Compared with individuals without depression, patients with depression have a considerably higher risk of the morbidity and mortality of diabetes, and are greatly affected by diabetes (6, 12). This linkage suggests shared potential biological mechanisms underlying depression and diabetes. It has been proposed that some pathological changes that participate in the process include abnormal hypothalamic-pituitary-adrenal axis (HPA axis) function, inflammation, environmental factors, and autonomic and neurohormonal dysregulation (13). Among these, the overactivation of innate immune system leading to a cytokine-mediated inflammatory response could target the brain, resulting in the increased risk of development of both depression and diabetes (14). Interleukin 6 (IL-6), a cytokine with wide immunological implications, was originally identified as a B cell differentiation factor (15). The interruption of IL-6 signaling plays an important role in the process of insulin resistance and the pathogenesis of T2DM (16). However, the role of IL-6 in insulin resistance seems to be more complex. Many studies have demonstrated that excessive IL-6 is involved in impaired insulin action on the liver and skeletal muscle of mice (17). In contrast, IL-6-deficient mice (IL-6^−^/^−^) showed that absence of IL-6 leads to the development of inflammation and insulin resistance in the liver (18), indicating that IL-6 might also play beneficial roles in the improvement of insulin sensitivity. Therefore, the role of IL-6 in the development of insulin resistance remains controversial, and might be tissue and activation phase dependent (19). Interestingly, emerging studies have shown that IL-6 is also involved in the regulation of energy metabolism in the central nervous system (20). Several mechanisms of IL-6-induced disruption of insulin signaling have been suggested. Particularly, IL-6 signaling could mediate JAK2-dependent regulation of signal transducer and activator of transcription protein 3 (STAT3). Insulin-induced phosphorylation of insulin receptor substrates 1 (IRS-1)/phosphoinositide 3-kinase (PI3K)-Akt cascades have been shown to be involved in the regulation of the glucose transporter 4 (GLUT4), thus participating in the process of glucose uptake and transport (21). However, whether depression susceptibility to hyperglycemia is associated with IL-6-mediated disruption of glucose homeostasis, is not clear.

Hence, it was hypothesized that stress-induced depressive disorder is likely to contribute to imbalance of glucose metabolism, and that cytokine IL-6-mediated disruption of glucose homeostasis signaling may participate in the process. In the current study, we first investigated the consequences of CUMS on depression-like behaviors. Further, the changes in the energy metabolism including peripheral glucose and lipid metabolism, body weight, and food intake were determined. Finally, we explored the mechanism of susceptibility of CUMS-induced depressive disorder to glucose metabolic disorder in hypothalamus based on IL-6-mediated glucose homeostasis signaling.

## Materials and Methods

### Animals

**Figure 1:**
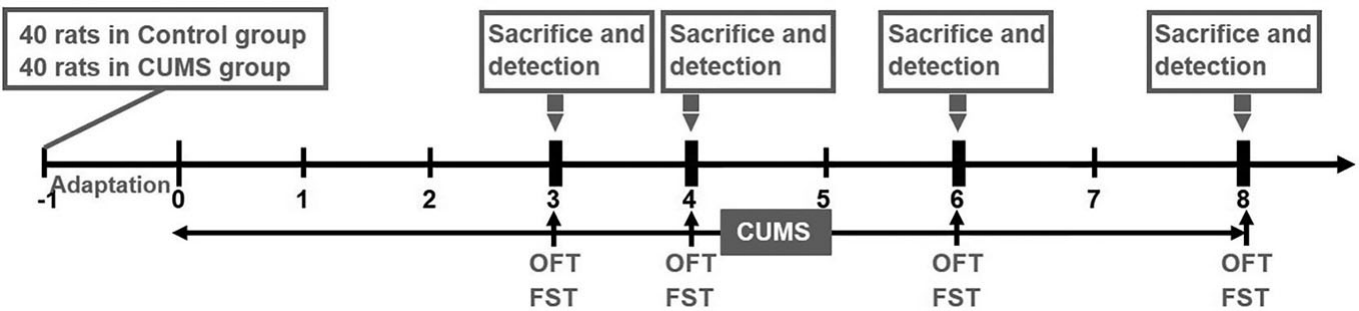
Experimental schedule. Prior to the experiment, a total of 80 rats (n = 40 in control group, n = 40 in CUMS group) were allowed a one-week adaptation period. Rats in the CUMS group received a daily CUMS stimulation, and behavior tests including OFT and FST were performed at various time points (3, 4, 6, 8 weeks). Subsequently, 10 rats from each of the two groups were sacrificed to detect the levels of blood glucose, blood lipids, proteins, and genes at the above time points.

A total of 80 male Sprague–Dawley rats (SCXK 2012-0001) weighing 180–200 g were obtained from Vital River Laboratory Animal Technology Limited Company (Beijing, China). The animals were maintained under standard laboratory conditions at room temperature of 25 ± 2°C and humidity 65 ± 5%. The standard 12 h light/dark cycle (light phase 6:00–18:00) was changed only in the course of the stress regime. Food and water were freely available to the rats except when food and water deprivation were applied as a stressor. After an acclimatization period of 7 days, an equal number of rats were randomly allocated to the Control group (n = 40) and CUMS group (n = 40). All experiments and animal care were approved by the Ethics Committee of China Academy of Chinese Medicine Sciences (No. 2016-0012) and were carried out in accordance with the National Institutes of Health Guide for the Care and Use of Laboratory Animals (National Research Council, 1996). The experimental design is shown in detail in **Figure 1**.

### CUMS Procedure

The rats were exposed to a CUMS as previously described with modifications (7). The rats were either exposed to CUMS for 3 to 8 weeks or kept as controls. The weekly stress regime consisted of food deprivation for 18 h followed by 1 h of restricted access to food, water deprivation for 18 h followed by 1 h exposure to an empty bottle, swimming in ice-cold water for 5 min, heat stress at 45°C for 5 min, white noise (85 dB) for 5 h, reversed light/dark cycle for 24 h, physical restraint for 3 h, and soiled bedding (200 ml water in 100 g sawdust bedding) for 17 h. The above stress regimes were randomized ensuring that each stressor was not repeated for two consecutive days.

### Behavioral Tests

#### Open Field Test (OFT)

OFT is a well-validated and commonly performed test for general locomotion and exploratory behavior. All animals were placed in the center of an open-field apparatus one by one to explore freely for 5 min before acclimatizing to the new environment. The activities of all the rats in the field were recorded by a video camera mounted above the arena. The total distance travelled and the time spent in the center of the field were recorded and analyzed using the Observer 5.0 software (Noldus, Netherlands) and EthoVision 14.0 software (Noldus, Netherlands).

#### Forced Swim Test (FST).

The animals were individually placed in a Plexiglas cylinder (50 cm height and 20 cm diameter) filled with water (30 cm depth and 20–25 °C) and allowed to swim for 15 min. Each rat was removed after 15 min, gently dried with a towel, and returned to its home cage. After 24 h, the rat was again placed in the cylinder filled with water and allowed to swim for 5 min. The immobility time, i.e., the time spent by the animal in floating in the water without struggling and making only movements necessary to keep its head above the water level, was recorded and analyzed.

### Measurement of Peripheral Glucose, Triglyceride (TG), Total Cholesterol (CHOL), High- and Low-Density Lipoprotein (HDL-C, LDL-C), Body Weight, and Food Intake

Morning fasting blood glucose, TG, CHOL, HDL-C, and LDL-C were determined using an automatic biochemical analyzer (Mindray, China).

The body weight during the acclimatization period was measured prior to the experiment as baseline weight and subsequently measured every week throughout the study. Food intake was monitored every 24 h and determined by subtracting the amount of remaining food including the spilled food at the bottom of the cage from their respective amount on the previous day.

### Hematoxylin–Eosin (HE) Staining and Immunohistochemical Analysis

The rats were anesthetized with 3% sodium pentobarbital by intraperitoneal injection and then fixed with 4% paraformaldehyde *via* transcardial perfusion. The liver, abdominal adipose tissues and hypothalamus were isolated and embedded in paraffin. Coronal sections of 5-μm thickness were cut using a rotary microtome (Leica, Wetzlar, Germany). The sections were stained with HE and the pathological changes were observed under a light microscope (Olympus, Tokyo, Japan).

The slices were first dewaxed and retrieved the antigens. Then, the endogenous peroxidases and the nonspecific staining in the tissues were successively blocked using a solution of 3% hydrogen peroxide for 10 min and 5% normal goat serum for 30 min at room temperature, respectively. Afterwards, the sections were incubated with anti-GLUT4 primary antibody (CST, #2213, diluted 1: 100) overnight at 4°C. The sections were next incubated in horseradish peroxidase-conjugated secondary antibody (diluted 1:1,000) for 2 h at room temperature and subsequently incubated in a DAB solution for 6 min. The images of the GLUT4-positive staining were analyzed using the Image-Pro Plus 6.0 software.

### Western Blotting (WB) Analysis

The rats were anesthetized with 3% sodium pentobarbital by intraperitoneal injection and their brains were rapidly removed on ice. Subsequently, the hypothalamic tissues were isolated from the brain in ice. All samples were immediately kept in liquid nitrogen and stored at −80°C until further analysis.

Total proteins were extracted from the rat hypothalamus tissues for the western blot analysis. A total of 30 µg protein was loaded on 10% SDS polyacrylamide gel and the resolved protein bands were subsequently transferred onto polyvinylidene difluoride membrane using a standard wet transfer system. The membranes were blocked with 5% nonfat milk at room temperature for 1 h, and subsequently incubated with corresponding primary antibodies [GLUT4, CST, #2213; IL-6, Abcam, #ab9324; P-STAT3 (Tyr705), CST, #9131; STAT3, ProteinTech, #10253-2-AP; P-IRS-1 (Ser307), CST, #2381; IRS-1, CST, #2390; P-PI3K (Tyr458/Tyr199), CST, #4228; PI3K, CST, #4249; Akt, ProteinTech, #60203-2-Ig; β-actin, ProteinTech, #66009-1-Ig] overnight at 4°C. Thereafter, the membranes were washed with TBST for 10 min (three times), incubated with appropriate Horse Radish Peroxidase (HRP) conjugated secondary antibodies at room temperature for 1 h, and then re-washed with TBST for 10 min (three times). The membranes were visualized with an enhanced chemiluminescence reagent (Bio-Rad, USA) on the ChemiDoc™ Imaging System (Bio-Rad, USA). The relative quantitation was calculated by normalization to β-actin.

### Real-Time Fluorescence Quantitative PCR (RT-qPCR) Analysis

Total RNA was isolated using Trizol reagent (Life Technologies, USA) according to a standard protocol. Reverse transcription was performed using a PrimeScript™ RT Master Mix (Takara, Cat.# RR036A) for cDNA synthesis on a Mastercycler^®^ nexus gradient (Eppendorf, Germany) according to the manufacturer’s instructions. All the reverse transcription reaction products were amplified with TB Green™ Advantage^®^ qPCR Premix kit (Takara, Cat. #639676) in a total volume of 25 μl on a CFX96 Real-time PCR System (Bio-Rad, USA) according to the two-step cycling parameters: 95°C for 30 s, 40 cycles of 95°C for 5 s, and 60°C for 30 s. The amplification reactions were performed in triplicates. The sequence of the GLUT4, IL-6, STAT3, IRS-1, PI3K, Akt, and GAPDH primers are listed in **Table 1**. Data were collected and expressed as values of threshold cycle. The relative expressions of the target genes were calculated by normalization to GAPDH expression.

**Table 1:** Sequence of oligonucleotides used for RT-qPCR.

Gene	Forward primer	Reverse primer	Annealing temperatures
GLUT4	AGCCAGCCTACGCCACCATAG	CAGCAGAGCCACCGTCATCAAG	62°C
IL-6	AGGAGTGGCTAAGGACCAAGACC	TGCCGAGTAGACCTCATAGTGACC	60°C
STAT3	CCAGTCGTGGTGATCTCCAACATC	CAGGTTCCAATCGGAGGCTTAGTG	60°C
IRS-1	AGCAACAGCAGCAGCAGTCTTC	ACTCTTCCGAGCCAGTCTCTTCTC	60°C
PI3K	AACTCGCCTCATAGCAGAGCAATG	TGGCACGCAGTCATGGTTGATC	59°C
Akt	GGCAGGAGGAGGAGACGATGG	TTCATGGTCACACGGTGCTTGG	60°C
GAPDH	CCATTCTTCCACCTTTGAT	TGGTCCAGGGTTTCTTACT	58°C

### Statistical Analysis

All data were analyzed using SPSS 20.0 software and expressed as mean ± standard deviation (SD). One-sample t test and repeated measures ANOVA were used when appropriate. The body weight data and food intake data were analyzed using Repeated-measures ANOVA to determine significant differences considering as the time and stress. A p-value <0.05 was considered statistically significant. All data were analyzed using GraphPad Prism 7.0.

## Results

### Provoked Depression-Like Behaviors After Exposure to CUMS in Rats

**Figure 2:**
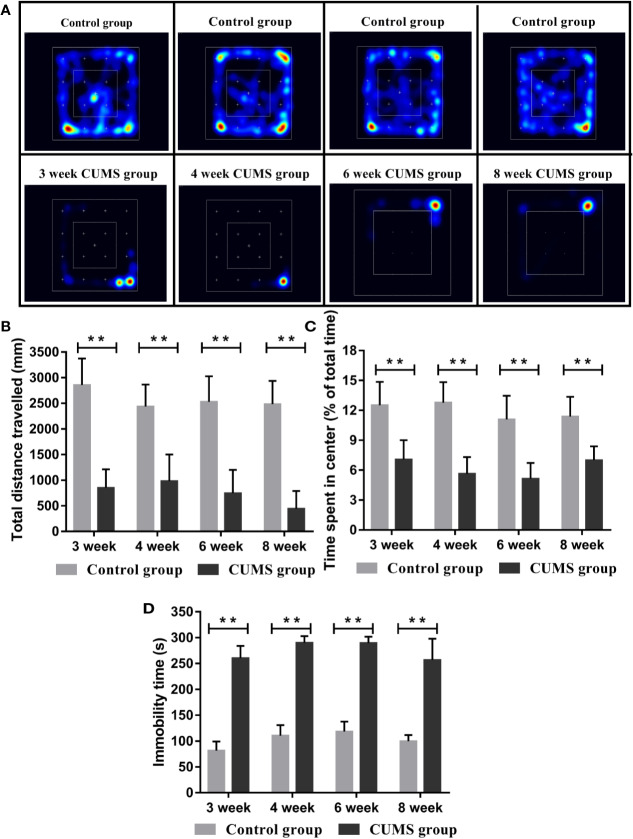
Effect of CUMS exposure on the depression-like behavior of rats. **(A)** Heat maps of the OFT. **(B)** Total distance travelled in the OFT. **(C)** Time spent in the center in OFT. **(D)** Immobility time in the FST. Data are presented as mean ± SD, n = 10; **p < 0.01 vs. Control group.

To assess the impact of CUMS on depression behaviors of rats, we measured the motor function, exploration activity, and immobility times of the CUMS group and the control group rats (16). As shown in **Figures 2A–C**, total distance travelled was significantly shorter and the time spent in the center was remarkably reduced in CUMS group as compared to the control group (p <0.01).

In the FST, rats exposed to CUMS stimulation for different time periods showed longer immobility time than rats in the control group (**Figure 2D**, p <0.01).

### Impaired Glucose and Lipid Metabolism and Decreased Body Weight and Food Intake in CUMS Exposed Rats

**Figure 3:**
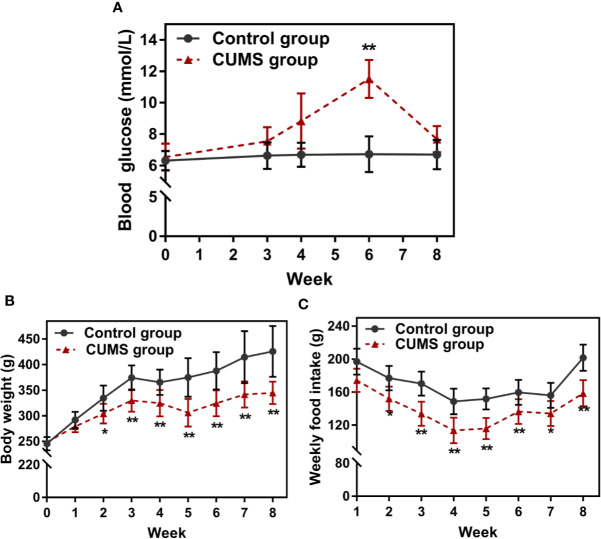
Effect of CUMS exposure on peripheral glucose, body weight, and food intake. **(A)** Fasting blood glucose. **(B)** Body weight. **(C)** Weekly food intake. Data are presented as mean ± SD, n = 10; *p < 0.05, **p < 0.01 vs. Control group.

Peripheral glucose and lipid metabolism, body weight, and food intake were compared between the CUMS group and the control group rats. As shown in **Figure 3A**, during CUMS lasting for 3 weeks, glucose levels increased slightly. However, the increase was statistically significant at week 6 when compared to the timed controls (p <0.01), however, a dramatical drop in glucose levels was reported after 6 weeks of consecutive CUMS stimulation.

As shown in **Table 2**, the impacts of our CUMS paradigm on lipid metabolism are contradictory and somewhat confusing. The TG levels were lower in animals with 3-week exposure to CUMS, however, a significant difference was observed only at 4-week CUMS exposure (p <0.05). Similarly, CHOL levels were significantly lower in CUMS-rats when compared to the control rats (p <0.05). The HDL-C levels in the CUMS group were lower than those, on the contrary, LDL-C levels were lower in 3-week group as compared with those of the control rats.

**Table 2:** Effects of CUMS exposure on TG, CHOL, HDL-C and LDL-C.

Group		TG (mmol/L)	CHOL (mmol/L)	HDL-C (mmol/L)	LDL-C (mmol/L)
Control group		0.318 ± 0.101	1.513 ± 0.240	1.159 ± 0.223	0.285 ± 0.049
CUMS group	3 weeks	0.255 ± 0.085	0.957 ± 0.239^**^	0.702 ± 0.170^**^	0.201 ± 0.058^**^
	4 weeks	0.222 ± 0.085^*^	1.241 ± 0.271^**^	0.888 ± 0.210^**^	0.254 ± 0.051
	6 weeks	0.295 ± 0.107	1.093 ± 0.198^**^	0.845 ± 0.145^**^	0.208 ± 0.046^**^
	8 weeks	0.343 ± 0.125	1.243 ± 0.290^**^	0.981 ± 0.231^*^	0.207 ± 0.042^**^

Data are presented as the mean ± SD, n = 10; *p < 0.05, **p < 0.01 vs. Control group.

The body weight and food intake were affected throughout as well as after the stress period. The body weight of control rats gradually increased with time, but the body weights of 2-week CUMS-exposed rats statistically decreased when compared with the control group (**Figure 3B**, p <0.05 at 2 weeks and p <0.01 at 3, 4, 5, 6, 7, 8 weeks). Consistently, CUMS made the animals consume less food weekly than corresponding control rats (**Figure 3C**, p <0.05 or p <0.01).

### Impaired Hepatocytes and Hypothalamic Neurons but Unaffected Adipocytes in CUMS Exposed Rats

**Figure 4:**
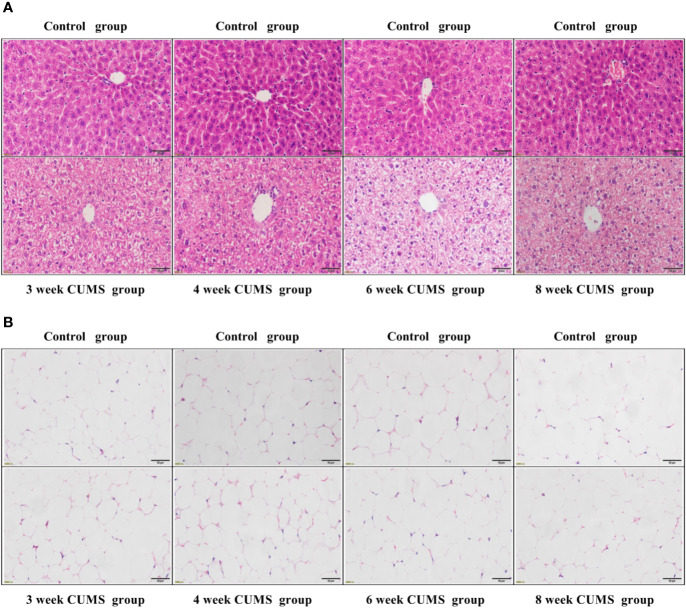
The influence of CUMS on hepatocytes and adipocytes using H&E staining. **(A)** Representative micrographs of rat liver tissues under a light microscope (scale bar = 50 μm, 400× magnification). **(B)** Representative micrographs of abdominal adipose tissues under a light microscope (scale bar = 50 μm, 400× magnification).

To evaluate the influence of CUMS on the function of hepatocytes, the rat liver tissues were stained using H&E staining. The results of liver histology are demonstrated in **Figure 4A**. In the control group, the hepatocytes showed well-preserved cytoplasm and clear nuclei, which were closely packed and regularly structured. The well-arranged hepatocytes with center nuclei were also seen in the CUMS group; however, enlarged cellular bodies and transparent cytoplasm with ballooning degeneration were also observed.

To confirm whether CUMS affected the size of adipocytes, the rat adipose tissues were stained using H&E staining. The histological analysis of adipose tissue is shown in **Figure 4B**. Our results showed that the rats exposed to CUMS displayed normal sized-adipocytes similar to that of the control group.

**Figure 5:**
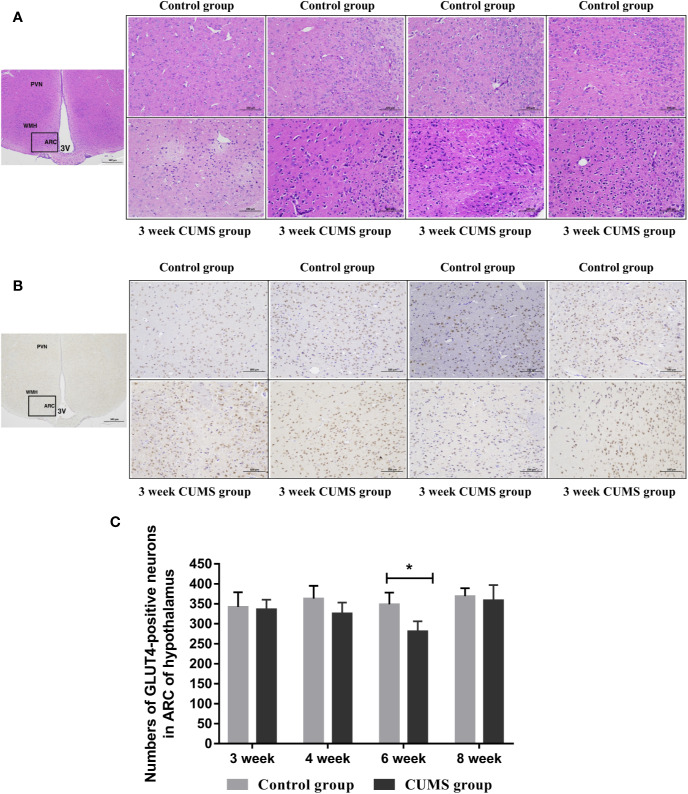
The influence of CUMS on histomorphology and GLUT4-postive neurons in hypothalamus of rats. **(A)** Representative micrographs of neurons in hypothalamus. **(B)** Representative micrographs of immunohistochemical staining for the GLUT4 proteins in the hypothalamus. The first micrographs was captured at low magnification (scale bar = 500 μm, 40x magnification); the remaining micrographs were captured at higher magnification (scale bar = 100 μm, 200x magnification), and observed the ARC of hypothalamus. **(C)** Quantitative analysis of the numbers of GLUT4-poistive neurons in the ARC of hypothalamus. PVN, paraventricular nucleus; VMH, ventromedialnucleus; ARC, arcuate nucleus; 3V, third ventricle. Data are presented as mean ± SD, n = 4 in immunohistochemical analysis; *p < 0.05 vs. Control group.

The effects of CUMS on the neuronal injury in arcuate nucleus (ARC) of hypothalamus were also determined using HE staining, as shown in **Figure 5A**. The normal neurons were stained in the control group, which displayed large, round cells with identifiable cell membranes, nuclei and discrete nucleoli. But, the CUMS-induced neurons exhibited cell shrinkage and pyknosis, small-sized and condensed nuclei, even or lack of nucleolus, indicating CUMS damaged the neurons in the ARC of hypothalamus.

### Reduced GLUT4 Expression in the Hypothalamus of Rats After 6-Week Exposure to CUMS

**Figure 6:**
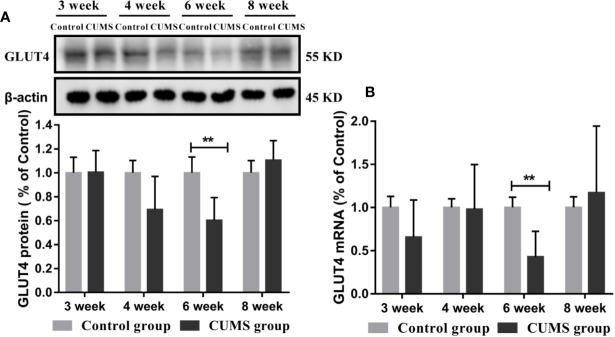
Effect of CUMS exposure on expression of GLUT4 in the hypothalamus of rats. **(A)** Protein levels of GLUT4 by western blot, the representative images for immunoblots are shown in the top panels, and quantitative data are shown in the bottom panels. **(B)** mRNA levels of GLUT4 by RT-qPCR. Data are presented as mean ± SD, n = 6; **p < 0.01 vs. Control group.

To study the effects of CUMS on glucose transport proteins in the brain, we measured GLUT4 protein and mRNA levels in rat hypothalamus. As shown in **Figures 5B, C**, a significant decrease in GLUT4-positive neurons was observed in the ARC of hypothalamus in rats after 6-week CUMS compared to those in the control group (P <0.05), whereas this change was no found in other stressed groups. Moreover, the results of GLUT4 proteins by WB analysis showed a consistent with the increased blood glucose levels by CUMS stimulation. As shown in **Figure 6A**, animals displayed a corresponding decrease in GLUT4 protein expression after CUMS exposure. Then, a reversed trend was observed following 8-week CUMS exposure. But, a statistically significant difference in GLUT4 protein expression was found only between 6-week CUMS-exposed rats compared with the control rats (p <0.01).

Similarly, rats exposed to CUMS for 6-weeks exhibited lower GLUT4 mRNA levels. However, a higher GLUT4 transcript levels were observed in rats exposed to CUMS for 8 weeks. Moreover, the data in 6-week CUMS group alone showed statistically significant difference (**Figure 6B**, p <0.001).

### Activated IL-6 and Impaired Insulin Signaling Pathway in the Hypothalamus of CUMS Rats

**Figure 7:**
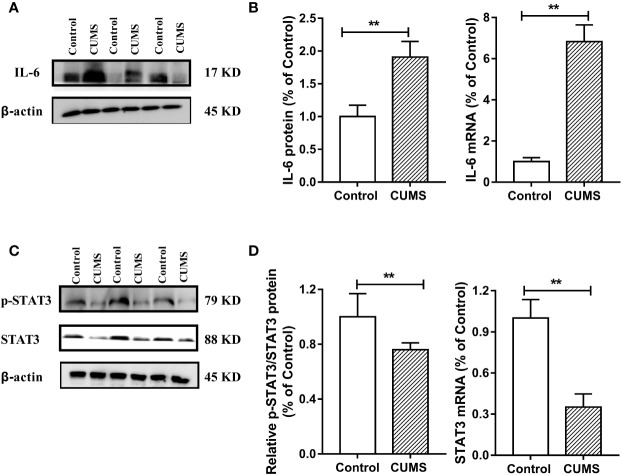
Effect of CUMS exposure on IL-6-mediated STAT3 signaling in the hypothalamus of rats. **(A)** The IL-6 protein representative images for immunoblots. **(B)** Protein and mRNA levels of IL-6. **(C)** The p-STAT3 and STAT3 protein representative images for immunoblots. **(D)** Relative p-STAT3/STAT3 protein and mRNA levels of STAT3. Data are presented as mean ± SD, n = 6; **p < 0.01 vs. Control group.

**Figure 8:**
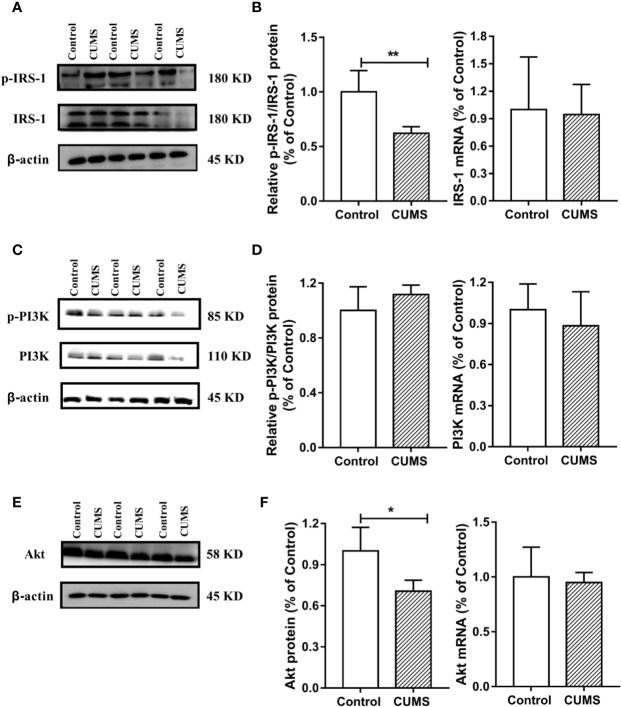
Effect of CUMS exposure on IL-6-mediated insulin signaling in the hypothalamus of rats. **(A)** The p-IRS-1 and IRS-1 protein representative images for immunoblots. **(B)** Relative p-IRS-1/IRS-1 protein and mRNA levels of IRS-1. **(C)** The p-PI3K and PI3K protein representative images for immunoblots. **(D)** Relative p-PI3K/PI3K protein and mRNA levels of PI3K. **(E)** The Akt protein representative images for immunoblots. **(F)** Protein and mRNA levels of Akt. Data are presented as mean ± SD, n = 6; *p < 0.05, **p < 0.01 vs. Control group.

To study the possible mechanism underlying susceptibility of CUMS-induced depression to hyperglycemia, we then detected the hypothalamic inflammatory cytokine-IL-6 and the insulin signaling pathway. As shown in **Figures 7A, B**, the protein and mRNA levels of IL-6 were signiﬁcantly increased in CUMS-exposed rats compared to the control group rats (p <0.01), suggesting that the expression of IL-6 was activated in the rats in response to CUMS. The results of insulin signaling pathway in the hypothalamus affected by CUMS were displayed in **Figures 7C, D**. The rats exposed to 6-week CUMS showed significantly lower levels of p-STAT3 and STAT3 in hypothalamus than Control group rats (p <0.01). In line with the trend of STAT3 signaling, impairment of insulin signaling was observed in the hypothalamus of CUMS-rats, with signiﬁcant decrease in the ratio of p-IRS1 to IRS-1 protein levels (p <0.01) and slight decrease in the mRNA levels of IRS-1 as compared to the control group (**Figures 8A, B**). Meanwhile, decreased expression of p-PI3K (p <0.01), PI3K (p <0.01), and Akt (p <0.05) genes were observed in the 6-week CUMS group (**Figures 8C, E**). However, there were no statistical differences in the ratio of p-PI3K to PI3K protein levels and the mRNA levels of PI3K and Akt between the two groups (p >0.05, **Figures 8D, F**).

## Discussion

**Figure 9:**
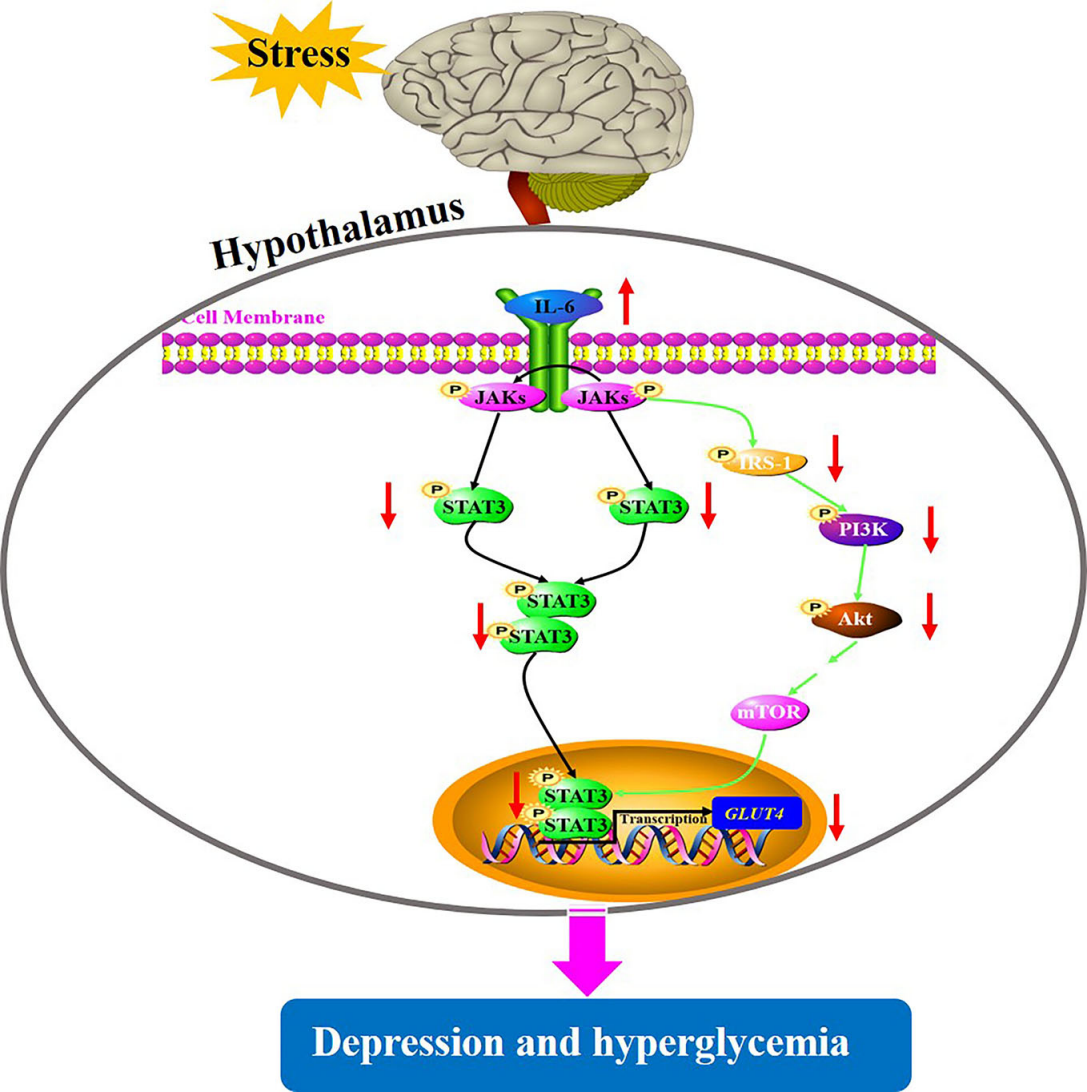
A putative mechanism underlying depression-like behavior susceptibility to hyperglycemia in rats, which is likely to be associated with the activation of IL-6-mediated inhibition of glucose homeostasis signaling in the hypothalamus.

Depression is a common mental health disorder with susceptibility to comorbid diabetes that has high morbidity, disability, and mortality. Accumulating evidences suggest that stress triggers neuroinflammation, which is most likely involved in the pathogenesis of depression comorbid with glucose intolerance (22). However, the roles of CUMS exposure, a common model to induce depression in rodents leading to glucose metabolic disorder, and of cytokine IL-6-mediated disruption of glucose homeostasis signaling in hypothalamus in the pathogenesis of depression comorbid with glucose intolerance, remain unknown. In the present study, rats exposed to CUMS for different periods of time showed susceptibility of depression-like behaviors to hyperglycemia. Importantly, the glucose metabolic disorder including high blood glucose and decreased GLUT4 levels in the hypothalamus were found only in rats exposed to 6-week CUMS stimulation. Furthermore, continuous exposure to CUMS for 6 weeks activated the inflammatory factor IL-6 in the hypothalamus of rats, resulting in the impairment of STAT3 and insulin signaling. This could have led to the reduction of GLUT4 in the hypothalamus of rats and consequently influence the peripheral glucose metabolism (**Figure 9**). Meanwhile, CUMS stimulation also provoked energy imbalance as evident by abnormal lipid metabolism and significant reduction in body weight and food intake with time.

Depression is a frequent comorbid condition in people with diabetes including both the major diabetes types. Clinical data has shown that one in every four people with T2DM are affected by depression (6). In turn, depression also increases the risk of the development of T2DM, and the subsequent risks of hyperglycemia and insulin resistance. Recent epidemiologic evidence showed that in both, types 1 and 2 diabetes, the depressive symptoms were associated with higher HbA1c, suggesting that depression was closely linked to hyperglycemia (23). In rodents, depression-like behavior could be induced by CUMS, and CUMS-induced depression comorbid glucose intolerant phenotypes were also reported in some studies (24, 25). On the contrary, another study reported lower blood glucose levels in depressive mice (26). Since the information available is inconsistent, this study attempted to reveal the association of CUMS-induced depression with changes in glucose levels. In line with previous reports (27), our study showed that CUMS stimulation for different time periods increased depression-like behaviors, which was shown by less exploration of a novel environment by the rats as evident by a decrease in the total distance travelled and time spent in the center of OFT, and increased response to hopelessness observed by a prolonged immobility time in the FST. Our results showed an initial gradual rise in blood glucose followed by a recovery trend with the extension of CUMS stimulation. However, a significant increase in blood glucose was shown only in 6-week CUMS-exposed rats. This phenomenon that the impact of stress on glucose levels in peripheral blood has been demonstrated in some parallel researches. The research found that glucose levels initially tended to drop for short-time stressed mice but were gradually increased with the prolonged stress time. Then, the peripheral hyperglycemia returned to baseline as the stress was continued (28). Furthermore, one of the primary reasons responsible for the trend may be related to the stress state, in which a temporary insulin resistance is driven by many complex factors such as counter-regulatory hormones like glucagon, cortisol, catecholamines, and the activation of pro-inflammatory factors like tumor necrosis factor-α (TNF-α), IL-6 (29, 30), consequently provoking the hyperglycemia. Therefore, the results indicated that the CUMS exposure, especially for 6 weeks, could induce depression-like behaviors and susceptibility to hyperglycemia in rats. However, unlike the clinical reports that have shown that an increase in the glucose intolerance is often accompanied by abnormal blood lipid metabolism, we obtained inconsistent results for blood lipid metabolism. Although the low HDL-C in CUMS-exposed rats partly reflected the characteristic of hyperlipidemia, decrease in TG, CHOL, and LDL-C levels were inconsistent with hyperlipidemia, and was probably linked to the reduction in food intake and body weight. Additionally, we also found that CUMS impaired the normal hepatocytes, one of the major sites of blood glucose homeostasis. Overall, our results provide evidence for a close association between CUMS-induced depression and hyperglycemia.

The linkage of depression and diabetes reflect that they may share a common biological origin. Chronic cytokine-mediated inflammatory response and overactivation of innate immunity have been extensively studied in the development of depression and diabetes (14, 31). IL-6 is a central cytokine in the regulation of innate immunity produced by a variety of cell types, such as immune cells, skeletal and smooth muscle cells, fibroblasts, microglial cells, astrocytes, and islet β-cells (32). Due to its broad tissue distribution, IL-6 is also involved in non-immune events including pathogeneses of insulin resistance, diabetes, and depression. Numerous studies have provided evidence that IL-6, *via* its actions on insulin sensitive tissues like adipose tissue, liver, skeletal muscle, and pancreatic islets, plays a significant role in the regulation of glucose metabolism (33). Similarly, treatment with IL-6 has been shown to have an effect on insulin signaling and translocation of GLUT4 in the adipose tissue and skeletal muscle, thus influencing glucose metabolism (34). Moreover, accumulating evidence from rodent and human studies suggests that IL-6 mediates the communication between peripheral and central nervous system, thereby playing a key role in the pathophysiology of depression (35, 36). Scientific data has provided evidence that a targeted approach to selectively inhibit IL-6 signaling may offer antidepressant effects (37). Our study further found that CUMS-induced depression and comorbid hyperglycemia activated IL-6 in the hypothalamus, suggesting that overactivation of innate IL-6 could play a crucial role in the pathogenesis of depression susceptibility to hyperglycemia, which is consistent with several previous reports.

STAT3 is one of the transcription factors regulating the production of the cytokine IL-6, IL-10, TNF-α, and IL-1β, which have been shown to be involved in depression (38). Importantly, IL-6/STAT3 signaling was shown to regulate the depressive behavior as well as the process of insulin resistance (39). Specifically, IL-6 binding to its receptor activates JAKs which subsequently phosphorylate STAT3, enabling its transport into the nucleus and downstream regulation of transcription of its target genes such as GLUT4, which are involved in glucose homeostasis. The insulin signaling pathway consisted of IRS-1/PI3K-Akt signaling which is a major mechanism underlying the development of diabetes (40). Activated JAK2 also binds and phosphorylates IRS-1, which regulates PI3K activity and subsequent phosphorylation of Akt, consequently participating in insulin signaling (41). Therefore, the dysfunction of glucose homeostasis signaling mediated by IL-6 eventually affects the transcription of GLUT4, partly elucidating the common biological mechanism of depression and diabetes. In this current study, GLUT4 mRNA and protein levels were significantly decreased by CUMS stimulation, possibly due to stimulated IL-6 expression, which induced the above signals. However, there are conflicting views on the influence of IL-6 on STAT3 in the regulation of depression as well as glucose homeostasis. For example, Sun-Ho Kwon and his colleagues knocked out STAT3 in CNS and reported that depression-related behaviors were regulated by cytokines; they further speculated that the inhibition of STAT3 could be a potential therapeutic strategy for depression (42). This result was consistent with many earlier findings (43, 44). On the other hand, conditional inactivation of IL-6 receptor or STAT3 has shown to prevent metabolic disturbance including obesity and insulin resistance (39). In contrast, it has been shown that the introduction of IL-6 improved obesity and decreased glucose tolerance by triggering the phosphorylation of STAT3 (19). These conflicts reports may be related to the characteristics of IL-6 depending on the tissue type. In our study, rats exposed to CUMS for 6 weeks displayed excessive activation of IL-6 and inhibition of STAT3 in the hypothalamus. Therefore, the suppression of STAT3 mediated by activated immune response could be closely associated with depression susceptibility to comorbid diabetes. Similarly, we also observed the IRS-1/PI3K-Akt insulin signal transduction in this study. Consistent with previous reports (45, 46), CUMS exposure inhibited the activation of IRS-1 and its cascade PI3K-Akt signaling, leading to reduced STAT3 in nucleus and consequently reduced transcription of GLUT4. These findings revealed that activation of IL-6 and suppression of its downstream signaling cascade including STAT3 and IRS-1/PI3K-Akt insulin signaling in the hypothalamus may be a potential shared mechanism underlying depression susceptibility to comorbid diabetes. However, there must be a feedback mechanism to explain why the active IL-6, instead of activating, inhibits its downstream signal transduction. Therefore, further studies are necessary to identify the mechanisms that play important regulatory roles in the IL-6-mediated decreased glucose homeostasis signaling in the hypothalamus of rats.

## Conclusion

In summary, to the best of our knowledge, this is the first study to show that exposure to CUMS for 6 weeks contributes to concurrent depression-like behaviors and hyperglycemia in rats. The mechanism underlying this model may be related to the activation of IL-6-mediated inhibition of glucose homeostasis signaling in the hypothalamus. Our data reported a decrease in IL-6 downstream signaling including STAT3 and IRS-1/PI3K-Akt insulin signaling in the hypothalamus. We believe that these findings will help to understand the pathology of depression comorbid diabetes from the view of neuroinflammation in brain and to develop novel therapeutic approaches.

## Data Availability Statement

All datasets generated for this study are included in the article/supplementary material.

## Ethics Statement

The animal study was reviewed and approved by Ethics Committee of China Academy of Chinese Medicine Sciences (No. 2016-0012).

## Author Contributions

XL and WQ designed, conducted the experiment and wrote the manuscript. NL helped conduct the animal experiments and behavior tests. XD, QM, YH, TW, and MS helped to collect and analyze the data. JC supervised the experiments and contributed to the final draft of the paper. All authors contributed to the article and approved the submitted version

## Funding

This work was supported by the National Natural Science Foundation of China (Nos. 81630104, 81973748), National Natural Science Foundation of China Youth Fund (Nos. 81904091, 81803998, 81703957), the Fundamental Research Funds for the Central Universities (No. 21619307), Medical Science and Technology Research Fund Project of Guangdong (No. A2019455), and Huang Zhendong Research Fund for Traditional Chinese Medicine of Jinan University.

## Conflict of Interest

The authors declare that the research was conducted in the absence of any commercial or financial relationships that could be construed as a potential conflict of interest.

## Abbreviations

T2DM, Type 2 diabetes mellitus; CUMS, Chronic unpredictable mild stress; HPA axis, Hypothalamic–pituitary–adrenal axis; IL-6, Interleukin 6; JAK2, Janus kinase 2; STAT3, Signal transducer and activator of transcription protein 3; IRS-1, Insulin receptor substrates 1; PI3K, Phosphoinositide 3-kinase; GLUT4, Glucose transporter 4; OFT, Open field test; FST, Forced swim test; TG, Triglyceride; CHOL, Total cholesterol; HDL-C, High density lipoprotein; LDL-C, Low density lipoprotein; WB, Western blotting; RT-qPCR, Real-time fluorescence quantitative PCR; SD, Standard deviation.
